# Wealth inequality as a predictor of HIV-related knowledge in Nigeria

**DOI:** 10.1136/bmjgh-2017-000461

**Published:** 2017-12-20

**Authors:** Lena Faust, Sanni Yaya, Michael Ekholuenetale

**Affiliations:** 1 Faculty of Health Sciences, University of Ottawa, Ottawa, Ontario, Canada; 2 School of International Development and Global Studies, University of Ottawa, Ottawa, Ontario, Canada; 3 The Women’s Health and Action Research Centre (WHARC), Benin City, Nigeria

**Keywords:** public health, HIV, AIDS

## Abstract

**Introduction:**

Considering the high state-level heterogeneity of HIV prevalence and socioeconomic characteristics in Nigeria, it is a relevant setting for studies into the socioeconomic correlates of HIV-related knowledge. Although the relationship between absolute poverty and HIV transmission has been studied, the role of wealth *inequality* in the dynamics of the HIV epidemic has yet to be investigated in Nigeria. The current study, therefore, investigates wealth inequality and other sociodemographic covariates as predictors of HIV-related knowledge, in order to identify subgroups of the Nigerian population that would benefit from HIV preventive interventions.

**Methods:**

This study used the nationally representative 2013 Nigerian Demographic and Health Survey (NDHS). HIV-related knowledge was computed as a total score based on HIV-related knowledge indicators in the NDHS, dichotomised using the sample median as the cut-off. Wealth inequality and other relevant sociodemographic variables were introduced into a logistic regression model based on their significance in bivariate analyses. ORs derived from the model were interpreted to identify risk groups for low HIV-related knowledge after adjusting for confounding factors.

**Results:**

The regression model indicated that individuals with lower literacy levels were almost twice as likely as literate respondents to have low HIV-related knowledge (adjusted OR (AOR): 1.95, 95% CI 1.85 to 2.05, P<0.001), and individuals in the upper wealth quintile were less than half as likely than those in the lower wealth quintile to have low HIV-related knowledge (AOR: 0.40, 95% CI 0.35 to 0.46, P<0.001). Women were also more than twice as likely as men to have low HIV-related knowledge at each level of wealth inequality. In addition, women were 80% less likely to have low mother-to-child transmission knowledge than men, but had over 1.5 times higher odds of having poor knowledge of HIV risk reduction measures. Ethnicity, religious affiliation, relationship status and residing in rural areas were additional significant predictors of HIV-related knowledge.

**Conclusion:**

HIV-related knowledge in this sample is generally low among women, those with low literacy levels, the poor, the unemployed, those residing in rural areas, those with traditional religious beliefs and those living in states with the highest wealth inequality ratios. The identification of these risk groups for low HIV-related knowledge facilitates the implementation of future evidence-based interventions among these groups in order to potentially reduce HIV transmission.

**Key questions**

**What is already known about this topic?**

- Nigeria exhibits high inter-regional heterogeneity in terms of HIV prevalence and socioeconomic characteristics.
- Although associations between absolute wealth and HIV transmission have received considerable attention in the existing literature, more recent Sub-Saharan African studies suggest that wealth inequality may be a more significant predictor of HIV transmission in the region.
- The importance of the improvement of HIV-related knowledge as a strategy for HIV prevention is recognised; however, risk groups for low HIV-related knowledge have not yet been investigated in the Nigerian context.

**What are the new findings?**

- HIV-related knowledge is generally low in this population, particularly with regard to the understanding of modes of mother-to-child transmission of HIV.
- Important predictors of low HIV-related knowledge in Nigeria include poverty, low literacy, ethnicity, religious affiliation, relationship status, being female under circumstances of wealth inequality and residing in rural areas.

**Recommendations for policy**

- Individuals with incomplete knowledge of HIV risk factors or transmission routes may underestimate their risk of infection, and thus represent a pertinent population subgroup for provider-initiated HIV testing and counselling (which have been shown to be highly feasible in the Nigerian context).
- Educational interventions covering the modes of transmission of HIV and preventive measures should be preferentially targeted at the above-mentioned identified risk groups for low HIV-related knowledge.
- In particular, the observed low knowledge of HIV risk reduction among women should be urgently addressed through the targeting of risk reduction interventions at women, with a particular focus on female-controlled preventive measures.
- These interventions should be adapted in terms of both content and mode of delivery to suit the needs of the target population, including for example the dissemination of verbal as opposed to written information to population subgroups with low literacy.

## Background

As Nigeria is both the most populous country in Africa^1 2^ as well as the country with the second highest number of people living with HIV in the world,^3^ studies into the epidemiology of HIV in the Nigerian context continue to be pertinent. As of 2013, 9% of the global burden of HIV cases were attributed to Nigeria alone,^3^ and in 2014 the country experienced an incidence of 220 000 new HIV cases.^4^ A total of 3.5 million people were estimated to be living with HIV in Nigeria as of 2015, and 180 000 deaths were attributed to HIV in the same year.^5^ Although the overall prevalence of HIV in Nigeria is estimated at 3.4%,^6^ there are wide disparities in HIV prevalence at the state level, ranging from 0.2% in Ekiti to 15.2% in Rivers.^7^

Many studies of HIV epidemiology have focused on absolute poverty as a risk factor for HIV infection^8^; however, in recent years, studies from Sub-Saharan Africa have reported that socioeconomic inequality is a stronger driver of HIV transmission than absolute measures of poverty or wealth.^9–12^ Despite Nigeria’s high overall HIV prevalence, its wide disparities in state-level HIV prevalence and the highly socioeconomically heterogeneous nature of its states, of the few studies that have investigated socioeconomic inequality as a driver of HIV transmission in Sub-Saharan Africa,^9–12^ none have done so in Nigeria.

Although low HIV-related knowledge (and consequently, low risk perception and potentially higher risk sexual practices) is considered an important contributory factor to the spread of HIV in Nigeria, detailed studies on how this knowledge differs among socioeconomic fault lines within the country are lacking.^6^ Given the country’s socioeconomic and cultural heterogeneity,^4^ determining the association of wealth inequality and other sociodemographic factors with HIV-related knowledge in Nigeria has the potential to provide valuable insight into the identification of population subgroups that may underestimate or be unaware of their risk of infection, and thus facilitate the evidence-informed design or modification of preventive interventions. Moreover, a previous study in Nigeria indicated high awareness of the existence of HIV, but low awareness of certain modes of transmission, particularly mother-to-child transmission (MTCT), suggesting that preventive behaviours regarding such transmission are low.^13^ The identification of sociodemographic groups with low HIV-related knowledge in Nigeria may therefore represent an initial step towards the eventual reduction of HIV transmission in the country.

The current study aimed to investigate the association between wealth inequality and other sociodemographic covariates with HIV-related knowledge in order to better understand the factors that drive the Nigerian HIV epidemic.

## Methods

### Data source

This study is based on the 2013 Nigerian Demographic and Health Survey (NDHS),^2^ a nationally representative survey of 38 948 women and 17 359 men in Nigeria, aged 15–49 years. The sampling procedure involved a three-stage stratification, in which respondents were first stratified by urban versus rural dwelling, and enumeration areas (EAs) were then selected randomly within each stratum. Lastly, 45 households within each EA were then selected for the survey using equal probability sampling. This three-stage sampling method was taken into account in the computation of survey weights, applied to ensure the representativeness of the sample with regard to the general population. Data for this study are derived from the individual female and male data sets, merged prior to data analysis. The response rates were 95% and 98% for the male and female data sets, respectively,^2^ resulting in a total weighted sample size of n=56 307.

### Conceptual framework

Prior to the examination of sociodemographic predictors of HIV-related knowledge in Nigeria, we present a theoretical framework within which to examine disparities in access to HIV-related knowledge and prevention resources across sociodemographic strata, particularly under circumstances of wealth inequality. The framework contains elements of both the biosocial and the health belief models. First, the health belief model,^14^ which has served as the conceptual basis of prior studies in the area of health-related knowledge and HIV,^15^ argues that individuals’ knowledge regarding a disease influences their perception of their risk of contracting it, and in turn their propensity to take preventive measures.^14^ Second, as underlined by the biosocial perspective and proponents of social medicine, socioeconomic status determines an individual’s ability to access health resources, and the socioeconomically marginalised therefore face structural barriers to accessing these resources, and are consequently at disproportionately high risk of suffering adverse health outcomes.^16–18^

Combining elements from the two models, we argue here that sociodemographic factors influence an individual’s access to health information—in this case, to HIV-related knowledge—and importantly an individual’s ability to ultimately transform this information into preventive action in order to actually secure better health outcomes. In addition, we underline that these sociodemographic factors do not necessarily operate in isolation, but rather that the confluence of multiple parameters of socioeconomic marginalisation may together determine individuals’ risk of low HIV-related knowledge, as well as their ability to use this knowledge to access the preventive health resources. For example, focusing on the relationship between gender and wealth inequality in the context of HIV risk, prior studies have noted that patterns of HIV risk among women compared with men differ across national, regional and economic fault lines, suggesting that the role of gender in HIV risk is influenced by prevailing sociodemographic or socioeconomic contexts.^19^

**Figure 1:**
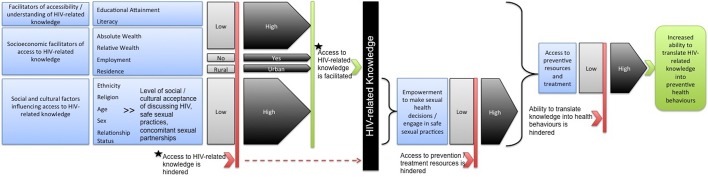
Theoretical framework for the determinants of HIV-related knowledge acquisition and translation (containing elements of the biosocial model and the health belief model).

Considering the potential relationship between wealth inequality, gender and HIV-related knowledge in Nigeria, this suggests that, being subject to the effects of both gender inequities and wealth inequality, women living in poverty may, first, have significantly reduced access to HIV education, thus lowering their HIV risk perception and their propensity to adopt preventive measures. Second, given that under circumstances of wealth inequality women may be driven to engage in transactional sex,^20 21^ our model underlines that their marginalisation as a result of both their gender and their poverty creates a scenario in which they are less likely to possess the knowledge to identify their risk of infection, the empowerment to put this knowledge into practice (ie, to negotiate preventive measures with their partner, such as condom use) and the means to actually access the required health resources (condoms, HIV testing, HIV information). Figure 1 provides a diagrammatic representation of the outlined theoretical framework.

### Outcome variable and predictors

The outcome variable, HIV-related knowledge, was computed as the sum of correct answers to HIV-related awareness and knowledge questions in the NDHS. For questions assessing HIV-related knowledge, answers were recoded as follows: correct answer=1, incorrect answer=0 and do not know=0 (see online supplementary appendix 1), and for questions assessing HIV-related awareness (questions 1–3, online supplementary appendix 1), aware=1 and unaware=0. The resulting total score was then dichotomised into high versus low HIV-related knowledge using the sample median score as the cut-off. Twelve questions were included in the HIV-related knowledge total score (online supplementary appendix 1), giving a highest possible score of 12. For a more detailed analysis of different areas of HIV-related knowledge, these 12 questions were then also separated into four knowledge domains (general HIV-related knowledge, knowledge of risk reduction measures, general knowledge of transmission routes and knowledge of MTCT), with total scores again dichotomised into low versus high according to the median for each domain. Despite the recognised statistical disadvantages of dichotomising a continuous variable,^22^ it remains a common approach for handling questionnaire or score data in the health sciences,^23–25^ and has also been used in a similar study investigating a Demographic and Health Surveyderived HIV-related knowledge score as the outcome variable.^26^ Although the mean was used in the aforementioned study, the median is used in the current study, as HIV-related knowledge scores in the current sample did not follow a standard distribution.

10.1136/bmjgh-2017-000461.supp1 Supplementary file 1

Based on the aforementioned conceptual framework derived from existing literature on potential predictors of HIV-related knowledge in Nigeria, the variables age, sex, absolute wealth, wealth inequality, educational attainment, literacy, employment status, relationship status, urban/rural dwelling, ethnicity and religion were considered in the investigation of correlates of HIV-related knowledge in this sample.

In the NDHS, national wealth quintiles are calculated based on an asset index of household goods (such as the ownership of livestock). As the continuous wealth score (based on the aforementioned asset index) arises from a principal component analysis and negative values are therefore possible, to calculate state-level wealth inequality, the raw wealth score was first transformed via additive transformation to yield only positive values. These were then sorted by state, and state-level wealth inequality was then computed as the ratio of the lower quintile over the upper quintile.

### Data analysis

Sociodemographic characteristics of the sample are reported using descriptive statistics. To optimise the representativeness of the sample, weights, as recommended by the DHS, were applied to descriptive analyses.

Bivariate analyses were carried out to investigate associations between each predictor variable and the dichotomous outcome variable HIV-related knowledge via the t-test for approximately normally distributed continuous variables and X^2^ analyses for categorical variables (including the categorical recodes of continuous variables).

An α value of 0.05 was considered indicative of statistical significance, except where appropriate Bonferroni corrections were applied.^27^ All analyses were conducted in SPPS V.24.0, except for the computation of state-level wealth inequalities, which was carried out in Stata V.14, because this computation required features not available in SPSS.

### Model variable selection and testing of assumptions

Variables yielding significant P values in X^2^ analysis were considered for inclusion in the initial logistic regression model, with wealth inequality as the focal predictor, adjusting for age, sex, ethnicity, religion, relationship status, rural/urban residence, literacy, absolute wealth and employment status. Variables were tested to determine whether the underlying assumptions of logistic regression were met.^28^

Testing the assumption of the linearity of continuous predictors, Box-Tidwell analysis^28^ demonstrated that none of the continuous variables were linearly related to the logit of HIV-related knowledge (all P<0.001) even after a Bonferroni correction was applied to the α value of 0.05,^27^ yielding an adjusted α value of 0.004. These continuous variables were therefore recoded into categorical variables.

To test for the absence of multicollinearity, a correlation matrix using Spearman’s correlation for ordinal*ordinal associations or Cramer’s V (φ_c_) for ordinal*nominal and nominal*nominal associations was carried out. Spearman’s correlation coefficients >0.7^29^ and φ_c_>0.5 were considered strong.^30^ Variables whose bivariate relationships had coefficients exceeding these thresholds were therefore considered highly correlated, and variables were thus removed from the model where appropriate.

Lastly, interaction terms were added based on conceptual relevance (eg, sex*wealth inequality).^20 21^ Based on the adjusted ORs (AORs) and corresponding CIs derived from this model, the odds of having low HIV-related knowledge in various sociodemographic strata were then interpreted to identify predictors of low HIV-related knowledge in the Nigerian population.

### Research ethics approval

The DHS programme obtains informed consent from participants and ensures their anonymity in accordance with the US Department of Health and Human Services regulations.

## Results

### Sample characteristics and HIV-related knowledge

The complete surveys constituted a total sample of n=56 307 (69.2% female), with respondents aged 15–49 years (mean age: 28.91, SD: 9.689). Of the sample, 39.4% had completed secondary education, 32.7% had no formal education at any level and only 10.7% had postsecondary education. The majority of the study population resided in a rural area (57.3%). Further sociodemographic characteristics of the study population are shown in table 1.

**Table 1:** Sociodemographic characteristics of the study population and bivariate analyses of associations with HIV-related knowledge

Sociodemographic variable	N (valid %) (unless otherwise indicated)	Per cent of demographic group with low† HIV-related knowledge	P value*
Total	56 307	41.0	
Age (years)			
15–24	21 088 (37.5)	46.1	<0.001
25–34	17 783 (31.6)	36.4	
35–49	17 436 (31.0)	39.6	
Mean age±SD	28.91±9.689		
(Range)	(15–49)		
Sex			
Female	38 948 (69.2)	43.8	<0.001
Male	17 359 (30.8)	35.0	
Highest level of educational attainment			
No formal education	18 414 (32.7)	64.1	<0.001
Primary education	9640 (17.1)	43.2	
Secondary education	22 208 (39.4)	33.0	
Postsecondary education	6044 (10.7)	12.5	
Literacy level (n=55 896)			
Illiterate, low literacy level or visually impaired	26 354 (47.1)	57.0	<0.001
Literate	29 542 (52.9)	28.8	
Employment status (n=56 025)			
Unemployed	18 720 (33.4)	47.4	<0.001
Employed	37 305 (66.6)	37.9	
Region of residence			
Urban	24 026 (42.7)	30.0	<0.001
Rural	32 281 (57.3)	49.1	
Ethnicity‡			
Fulani	3518 (6.2)	65.5	<0.001
Hausa	15 417 (27.4)	55.6	
Ibibio	1261 (2.2)	32.1	
Igbo	7967 (14.1)	31.4	
Ijaw	1097 (1.9)	23.4	
Yoruba	7823 (13.9)	29.3	
Other	19 225 (34.1)	38.7	
Religion			
Catholicism	6329 (11.2)	32.4	<0.001
Other Christian	20 102 (35.7)	30.1	
Islam	29 057 (51.6)	52.5	
Traditionalism	521 (0.9)	62.3	
Other or none	298 (0.5)	39.7	
Relationship status			
Never in union	17 704 (31.4)	38.4	<0.001
Currently in union or cohabiting	36 552 (64.9)	42.5	
Formerly in union or cohabiting	2051 (3.6)	38.1	
National wealth quintile			
Lowest 20th	9994 (17.7)	69.1	<0.001
20th–40th	10 420 (18.5)	53.7	
40th–60th	10 824 (19.2)	41.2	
60th–80th	11 827 (21.0)	32.2	
Highest 20th	13 242 (23.5)	21.7	
State-level wealth inequality ratio category			
<1.50	8012 (14.2)	28.5	<0.001
1.50–1.79	20 629 (36.6)	41.0	
1.80–2.19	25 578 (45.4)	46.0	
>2.19	2088 (3.7)	39.0	
HIV-related knowledge score (median (IQR))§			
(n=51 530)	9 (7–11)		

*P values determined by X^2^ tests.

†Below sample median (<9).

‡Specific ethnic groups shown are those with >1000 members.

§Highest possible total score=12.

The study population had limited HIV-related knowledge, with a median HIV-related knowledge score of 9 (IQR: 7–11) out of 12. Notably, 6.7% of the sample had never heard of AIDS, and only 11.5% of respondents correctly answered all 12 HIV-related knowledge questions. Knowledge of the possible modes of MTCT of HIV was average in this sample, with 58.4%, 58.2% and 69.0% of the respondents reporting knowing that HIV can be transmitted during pregnancy, delivery or breast feeding, respectively. Bivariate analyses for the associations of sociodemographic characteristics with HIV-related knowledge are provided in table 1, and proportions of correct responses for each question are shown in table 2.

**Table 2:** Proportion of respondents with correct knowledge or awareness of HIV-related knowledge indicators in each knowledge domain

Knowledge area	Nigerian Demographic and Health Survey HIV-related knowledge question	N correct or N aware (valid %)
General HIV-related knowledge	Has heard of AIDS* (n=56 285)	52 509 (93.3)
	A healthy looking person can have HIV (n=52 155)	39 470 (81.3)
	Knows a place to get HIV testing* (n=52 434)	36 136 (68.9)
Knowledge of HIV risk reduction	To reduce the risk of getting HIV: have one sex partner only, who has no other partners (n=52 428)	44 817 (85.5)
	To reduce the risk of getting HIV: always use condoms during sex (n=52 399)	35 353 (67.5)
	Knows a source for condoms* (n=56 122)	32 471 (57.9)
Knowledge of modes of transmission	Can contract HIV from mosquito bite (n=52 472)	36 975 (70.5)
	Can contract HIV by sharing food with person who has AIDS (n=52 420)	42 620 (81.3)
	Can contract HIV by witchcraft or supernatural means (n=52 374)	36 328 (69.4)
Knowledge of mother-to-child transmission	HIV can be transmitted during pregnancy (n=52 482)	30 673 (58.4)
	HIV can be transmitted during delivery (n=52 472)	30 547 (58.2)
	HIV can be transmitted by breast feeding (n=52 476)	36 221 (69.0)

*Questions indicating awareness rather than knowledge, and coded accordingly (see Methods section).

### Bivariate analyses of associations of sociodemographic characteristics with HIV-related knowledge

Bivariate analyses of the association of all categorical independent variables with HIV-related knowledge were found to be significant via X^2^ tests (P<0.001). Based on these analyses, it was found that the proportion of low HIV-related knowledge was significantly higher among respondents aged 15–24 years than among older age groups, among women than men, among the unemployed than the employed, and among those living in rural compared with urban areas. In addition, the proportion of respondents with low HIV-related knowledge decreased with each additional level of educational attainment, and similarly a decrease in the proportion of respondents with low HIV-related knowledge was observed at each level from the lower 20th to the upper 20th wealth quintiles. Lastly, proportions of low HIV-related knowledge also differed significantly among ethnic and religious groups, literacy levels, and wealth inequality categories. Results of the bivariate analyses are provided in table 1.

t-Tests were carried out for the continuous variables age and wealth inequality, both demonstrating significant differences by HIV-related knowledge category (age: t=−14.429, P<0.001; wealth inequality: t=23.191, P<0.001).

### Multicollinearity testing

Due to its high collinearity with literacy (r_s_=0.877), educational attainment was removed from the model, as literacy may be a more accurate representation of an individual’s understanding of HIV-related information than educational level, as the latter may be more subjective, given that the quality of education at each level or the classification of these levels may vary across the country. Additionally, urban/rural residence was found to be strongly associated with wealth quintile (df=1, φ_c_= 0.586); however, in this case, both variables were retained in the model, given that the effects of urban/rural residence on HIV-related knowledge may in some ways be independent of the effects of wealth on HIV-related knowledge, representing an additional and unique barrier in terms of access to information, for example through lower coverage of HIV awareness campaigns in rural compared with urban areas, regardless of wealth status. Educational attainment was therefore the only variable removed in response to multicollinearity testing.

### Predictors of HIV-related knowledge in Nigeria

Literacy level, employment status, relationship status, age category, urban/rural residence, sex, ethnicity, wealth quintile, state-level wealth inequality ratio category and religion were all predictive of HIV-related knowledge, and the final logistic regression model was found to be statistically significant (P<0.001), correctly predicting the HIV-related knowledge category of 68.1% of all cases. In comparison, the null model, containing only the constant, correctly classified 59.0% of the cases.

Respondents living in urban areas had approximately 20% lower odds of having low HIV-related knowledge compared with residents of rural areas (AOR: 0.83, 95% CI 0.79 to 0.87, P<0.001) (table 3). Moreover, respondents in the youngest age category (15–24 years) had significantly higher odds of having low HIV-related knowledge than respondents in the oldest age category (35–49 years) (AOR: 1.35, 95% CI 1.22 to 1.49, P<0.001). In addition, respondents reporting being Traditionalist had significantly higher odds of low HIV-related knowledge than any other religious category (Catholics: AOR: 0.48, 95% CI 0.38 to 0.60, P<0.001; other Christians: AOR: 0.49, 95% CI 0.39 to 0.60, P<0.001). Respondents with low literacy levels were almost twice as likely as literate respondents to have low HIV-related knowledge (AOR: 1.95, 95% CI 1.85 to 2.05, P<0.001).

**Table 3:** Logistic regression model for the prediction of HIV-related knowledge in Nigeria

Variable	AOR	95% CI		P value	OR	95% CI		P value
		Lower	Upper			Lower	Upper	
Ethnicity								
(Yoruba)†								
Other	0.94	0.87	1.00	0.066	1.53	1.44	1.62	0.000
Fulani	1.15	1.02	1.29	0.020	4.58	4.17	5.03	0.000
Hausa	0.97	0.89	1.05	0.407	3.03	2.85	3.22	0.000
Ibibio	0.96	0.83	1.11	0.572	1.14	1.00	1.30	0.047
Igbo	1.00	0.92	1.09	0.991	1.10	1.03	1.18	0.005
Ijaw	0.59	0.52	0.68	0.000	0.74	0.66	0.82	0.000
Religion								
(Traditionalism)†								
Other or none	0.58	0.40	0.83	0.003	0.40	0.29	0.55	0.000
Catholicism	0.48	0.38	0.60	0.000	0.29	0.23	0.36	0.000
Other Christian	0.49	0.39	0.60	0.000	0.26	0.21	0.32	0.000
Islam	0.72	0.57	0.89	0.003	0.67	0.54	0.82	0.000
Region of residence								
(Rural)†								
Urban	0.83	0.79	0.87	0.000	0.44	0.43	0.46	0.000
National wealth quintile								
(Lower 20th)†								
20th–40th	0.66	0.59	0.74	0.000	0.52	0.49	0.55	0.000
40th–60th	0.58	0.51	0.65	0.000	0.31	0.29	0.33	0.000
60th–80th	0.52	0.46	0.59	0.000	0.21	0.20	0.23	0.000
Upper 20th	0.40	0.35	0.46	0.000	0.12	0.12	0.13	0.000
Relationship status								
(Never in union)†								
Currently in union or cohabiting	0.78	0.74	0.83	0.000	1.19	1.14	1.23	0.000
Formerly in union or cohabiting	0.78	0.70	0.88	0.000	0.99	0.90	1.09	0.845
Current employment status								
(Employed)†								
Unemployed	1.28	1.22	1.34	0.000	1.47	1.42	1.53	0.000
Sex								
(Male)†								
Female	0.77	0.57	1.04	0.090	1.45	1.39	1.50	0.000
State-level wealth inequality ratio category								
(>2.19)†								
<1.5	1.09	0.85	1.40	0.489	0.63	0.55	0.72	0.000
1.5–1.79	1.05	0.83	1.32	0.689	1.09	0.96	1.24	0.200
1.80–2.19	0.91	0.73	1.15	0.427	1.33	1.17	1.52	0.000
Age group								
(35–49)†								
15–24	1.35	1.23	1.49	0.000	1.30	1.25	1.36	0.000
25–34	1.02	0.94	1.12	0.609	0.87	0.83	0.91	0.000
Literacy level								
(Literate)†								
Low literacy or visually impaired	1.95	1.85	2.05	0.000	3.27	3.16	3.40	0.000
Age*sex								
(35–49*male)†								
15–24*female	0.94	0.85	1.04	0.252	1.50	1.44	1.57	0.000
25–34*female	0.82	0.74	0.91	0.000	1.00	0.96	1.05	0.949
Sex*wealth quintile								
(Male*lower 20th)†								
Female*20th–40th	0.94	0.82	1.08	0.385	1.74	1.64	1.84	0.000
Female*40th–60th	0.78	0.68	0.89	0.000	0.99	0.94	1.05	0.762
Female*60th–80th	0.65	0.57	0.75	0.000	0.63	0.59	0.66	0.000
Female*upper 20th	0.56	0.49	0.65	0.000	0.34	0.32	0.36	0.000
Sex*wealth inequality ratio category								
(Male*>2.19)†								
Female*<1.5	2.36	1.74	3.21	0.000	0.76	0.71	0.81	0.000
Female*1.5–1.79	2.51	1.87	3.35	0.000	1.46	1.39	1.52	0.000
Female*1.80–2.19	2.58	1.93	3.44	0.000	1.84	1.76	1.93	0.000

Variables adjusted for ethnicity, religion, region of residence, national wealth quintile, relationship status, employment status, sex, state-level wealth inequality ratio, age group, literacy level, age*sex, sex*wealth quintile, sex*wealth inequality ratio.

†Reference category.

AOR, adjusted OR; OR, unadjusted (crude) odds ratio.

Respondents in the upper 20th wealth quintile were more than two times less likely to have low HIV-related knowledge as those in the lower 20th wealth quintile (AOR: 0.40, 95% CI 0.35 to 0.46, P<0.001), and the odds of low HIV-related knowledge rose significantly (P<0.001) in each wealth category as wealth decreased.

**Figure 2:**
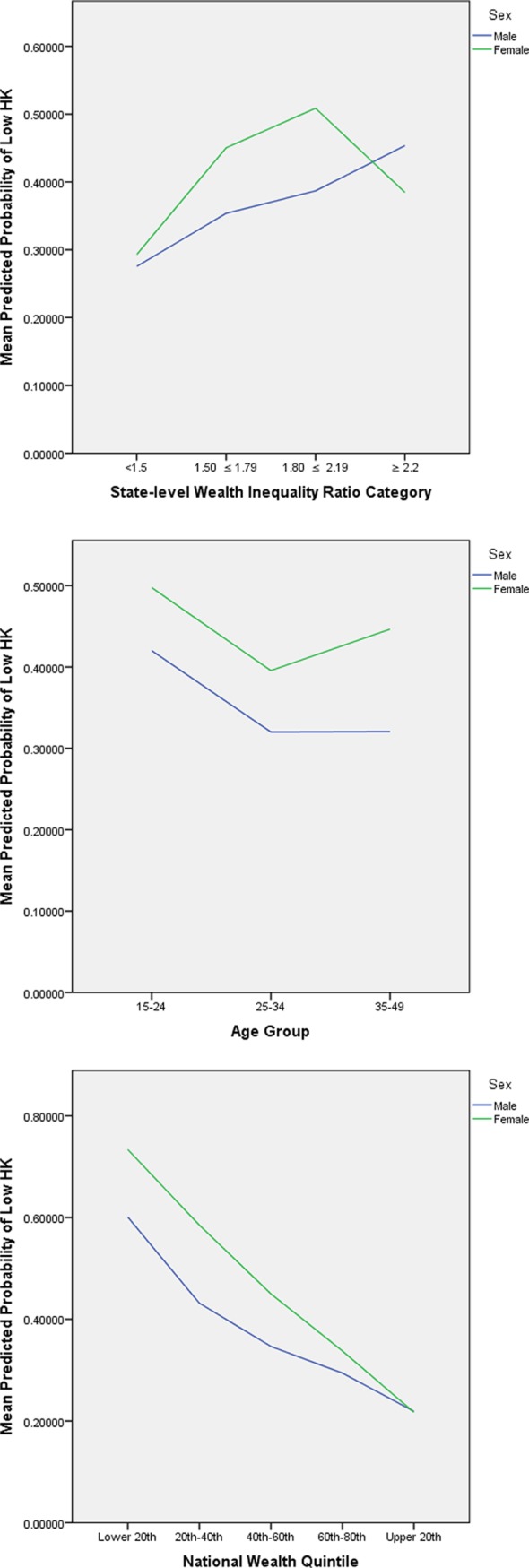
Interaction plots: predicted probabilities of low HIV-related knowledge (HK) by age*sex, sex*absolute wealth and sex*wealth inequality.

Sex alone was not a significant predictor of HIV-related knowledge overall in this model (P=0.09); however, interaction terms show that women were more than twice as likely as men to have low HIV-related knowledge in lower wealth inequality categories than men in the highest wealth inequality category (figure 2). Across the first three wealth inequality categories, the sex*wealth inequality interaction plot demonstrates a steeper rise in the probability of low HIV-related Knowledge for women than for men as wealth inequality increases, and the overall probability of having low HIV-related knowledge is higher for women than for men at each level of wealth inequality. However, the probability of low HIV-related knowledge decreases considerably from the second highest to highest wealth inequality categories among women, even falling below that of men.

### Predicting specific domains of HIV-related knowledge: MTCT, other routes of transmission and risk reduction

The analyses of the odds of low knowledge of MTCT (table 4) revealed that women are 80% less likely to have low knowledge of MTCT of HIV than men (AOR: 0.23, 95% CI 0.17 to 0.31, P<0.001), but over 1.5 times as likely as men to have low knowledge of risk reduction measures (AOR: 1.58, 95% CI 1.14 to 2.18, P<0.006). The odds of low knowledge of MTCT are highest among the Fulani and lowest among the Ijaw ethnic groups (Fulani: AOR: 1.13, 95% CI 1.01 to 1.26, P<0.028; Ijaw: AOR: 0.54, 95% CI 0.47 to 0.61, P<0.001).

**Table 4:** Logistic regression for the prediction of four HIV-related knowledge domains

Variable	Knowledge of mother-to-child transmission				Knowledge of risk reduction				General knowledge of HIV				Understanding of routes of transmission			
	AOR	95% CI		P value	AOR	95% CI		P value	AOR	95% CI		P value	AOR	95% CI		P value
		Lower	Upper			Lower	Upper			Lower	Upper			Lower	Upper	
Ethnicity																
(Yoruba)†																
Other	0.96	0.89	1.02	0.196	0.97	0.88	1.06	0.514	0.82	0.77	0.88	0.000	1.04	0.98	1.11	0.234
Fulani	1.13	1.01	1.26	0.028	1.53	1.34	1.73	0.000	1.15	1.03	1.29	0.016	0.73	0.66	0.82	0.000
Hausa	0.91	0.84	0.99	0.024	1.32	1.19	1.46	0.000	1.44	1.33	1.57	0.000	0.44	0.41	0.48	0.000
Ibibio	0.92	0.79	1.06	0.232	1.17	0.98	1.41	0.084	0.66	0.57	0.77	0.000	1.43	1.25	1.64	0.000
Igbo	0.96	0.88	1.04	0.332	1.41	1.26	1.57	0.000	2.22	2.04	2.41	0.000	0.76	0.70	0.82	0.000
Ijaw	0.54	0.47	0.61	0.000	0.87	0.75	1.03	0.098	0.62	0.55	0.71	0.000	0.98	0.88	1.09	0.674
Religion																
(Traditionalism)†																
Other or none	0.79	0.56	1.12	0.187	0.70	0.47	1.03	0.067	0.70	0.49	1.00	0.048	0.81	0.58	1.14	0.226
Catholicism	0.73	0.59	0.91	0.004	0.36	0.28	0.45	0.000	0.62	0.49	0.77	0.000	0.84	0.68	1.05	0.128
Other Christian	0.70	0.57	0.86	0.001	0.47	0.38	0.59	0.000	0.61	0.49	0.75	0.000	0.78	0.63	0.96	0.020
Islam	1.04	0.85	1.29	0.692	0.77	0.61	0.96	0.021	0.79	0.63	0.98	0.033	0.74	0.59	0.91	0.005
Region of residence																
(Rural)†																
Urban	0.92	0.88	0.97	0.001	0.80	0.75	0.85	0.000	0.81	0.77	0.85	0.000	0.93	0.89	0.98	0.003
National wealth quintile																
(Lower 20th)†																
20th–40th	0.77	0.69	0.86	0.000	0.67	0.58	0.77	0.000	0.73	0.65	0.82	0.000	0.80	0.72	0.90	0.000
40th–60th	0.70	0.63	0.79	0.000	0.45	0.38	0.52	0.000	0.67	0.59	0.75	0.000	0.83	0.74	0.93	0.002
60th–80th	0.73	0.65	0.82	0.000	0.36	0.30	0.42	0.000	0.57	0.50	0.64	0.000	0.70	0.62	0.79	0.000
Upper 20th	0.75	0.66	0.85	0.000	0.35	0.29	0.42	0.000	0.40	0.35	0.46	0.000	0.46	0.41	0.52	0.000
Relationship status																
(Never in union)†																
Currently in union or cohabiting	0.74	0.70	0.79	0.000	0.80	0.74	0.86	0.000	0.85	0.80	0.90	0.000	0.99	0.94	1.05	0.749
Formerly in union or cohabiting	0.81	0.72	0.91	0.000	0.67	0.58	0.76	0.000	0.83	0.74	0.92	0.001	1.02	0.92	1.13	0.707
Employment status																
(Employed)†																
Unemployed	1.22	1.17	1.28	0.000	1.17	1.11	1.23	0.000	1.48	1.41	1.55	0.000	0.90	0.86	0.94	0.000
Sex																
(Male)†																
Female	0.23	0.17	0.31	0.000	1.58	1.14	2.18	0.006	1.67	1.23	2.25	0.001	0.36	0.26	0.51	0.000
State-level wealth inequality ratio																
(>2.19)†																
<1.5	0.31	0.24	0.40	0.000	0.72	0.53	0.98	0.034	1.08	0.84	1.38	0.569	3.20	2.49	4.12	0.000
1.5–1.79	0.31	0.25	0.40	0.000	0.64	0.49	0.83	0.001	0.99	0.78	1.25	0.906	3.01	2.37	3.82	0.000
1.80–2.19	0.39	0.31	0.50	0.000	0.46	0.35	0.60	0.000	1.12	0.89	1.41	0.337	2.63	2.07	3.33	0.000
Age group																
(35-49)†																
15–24	1.08	0.99	1.19	0.101	1.37	1.20	1.55	0.000	1.45	1.31	1.59	0.000	1.46	1.34	1.60	0.000
25–34	1.02	0.94	1.11	0.608	0.83	0.72	0.94	0.005	0.98	0.90	1.07	0.685	1.07	0.99	1.16	0.106
Literacy level																
(Literate)†																
Illiterate, low literacy or visually impaired	1.37	1.30	1.44	0.000	1.95	1.84	2.08	0.000	1.90	1.80	2.00	0.000	1.49	1.42	1.57	0.000
Age*sex																
(35–49*male)†																
15–24*female	1.17	1.06	1.29	0.001	0.81	0.71	0.92	0.001	0.80	0.72	0.88	0.000	0.86	0.78	0.94	0.001
25–34*female	0.91	0.82	1.00	0.060	0.98	0.84	1.13	0.745	0.86	0.77	0.95	0.004	0.98	0.89	1.08	0.697
Sex*wealth quintile																
(Male*lower 20th)*																
Female*20th–40th	0.91	0.80	1.05	0.194	1.05	0.90	1.22	0.549	0.81	0.70	0.93	0.003	1.14	0.99	1.30	0.061
Female*40th–60th	0.74	0.65	0.85	0.000	1.25	1.06	1.48	0.009	0.67	0.58	0.77	0.000	1.05	0.92	1.20	0.465
Female*60th–80th	0.60	0.52	0.68	0.000	1.29	1.08	1.54	0.006	0.66	0.57	0.76	0.000	0.98	0.86	1.12	0.797
Female*upper 20th	0.51	0.45	0.59	0.000	1.03	0.85	1.25	0.753	0.64	0.55	0.74	0.000	1.20	1.05	1.37	0.008
Sex*wealth inequality ratio category																
(Male*>2.19)†																
Female*<1.5	6.30	4.63	8.57	0.000	1.35	0.95	1.93	0.095	1.43	1.05	1.93	0.022	2.44	1.74	3.43	0.000
Female*1.5–1.79	5.15	3.84	6.90	0.000	1.89	1.38	2.59	0.000	1.52	1.14	2.03	0.004	2.50	1.80	3.47	0.000
Female*1.80–2.19	5.02	3.75	6.72	0.000	2.16	1.58	2.95	0.000	1.21	0.91	1.61	0.198	2.44	1.76	3.38	0.000

Variables adjusted for ethnicity, religion, region of residence, national wealth quintile, relationship status, employment status, sex, state-level wealth inequality ratio, age group, literacy level, age*sex, sex*wealth quintile, sex*wealth inequality ratio.

†Reference category.

AOR, adjusted OR.

The odds of low knowledge of MTCT were approximately 20%–30% lower in the four upper wealth quintiles compared with the lowest quintile (AORs (range): 0.70–0.77, all P<0.001), and the odds of low knowledge of other routes of transmission decreased by 20% to more than 50% with increasing wealth quintiles. Similarly, those in the 20th–40th wealth quintile are more than 30% less likely to have low knowledge of risk reduction than those in the lowest quintile, and these odds of low knowledge decrease with each increase in wealth quintile (all P<0.001).

In states with lower wealth inequality, the odds of low knowledge of MTCT were approximately 60%–70% lower than in states in the highest wealth inequality category. Furthermore, the odds of low knowledge of risk reduction are between approximately 30% to more than 50% lower in the four lower wealth inequality categories compared with the highest wealth inequality category.

Additionally, the odds of low knowledge of risk reduction are 20% lower among urban dwellers in comparison to rural dwellers (AOR: 0.80, 95% CI 0.75 to 0.85, P<0.001), while the odds of knowledge of MTCT and other transmission modes are similar among rural compared with urban dwellers. On the other hand, with regard to age, respondents in the youngest age group (15–24 years) have greater odds of low knowledge than older respondents across all knowledge domains, and should thus be a priority target for HIV education. Lastly, those with low literacy levels were approximately twice as likely to have low knowledge of risk reduction and 1.5 times as likely to have low knowledge of modes of transmission in comparison to the literate, suggesting that individuals with low literacy face significant barriers to the acquisition of HIV-related knowledge.

**Figure 3:**
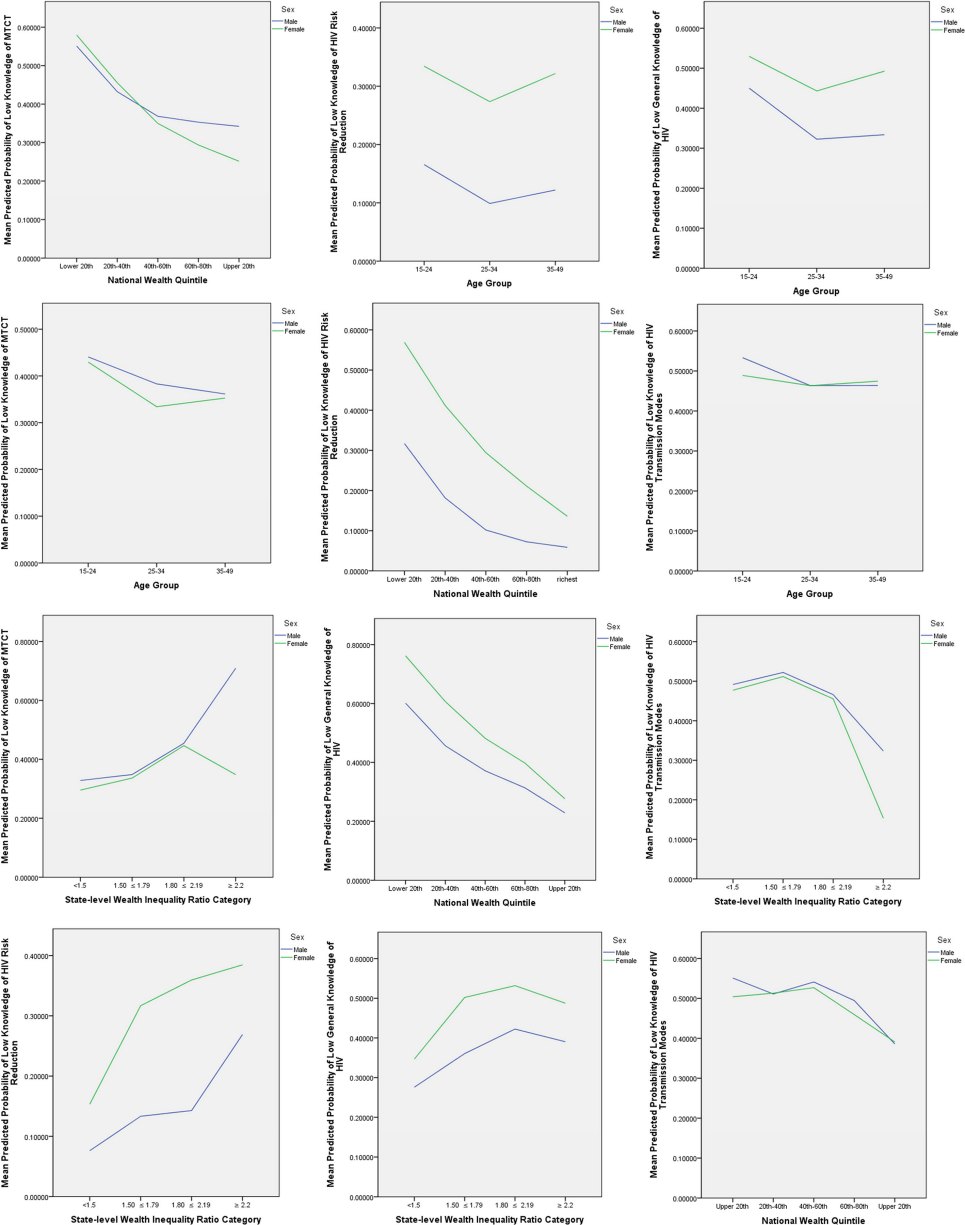
Interaction plots: predicted probabilities of low knowledge across four knowledge domains. MTCT, mother-to-child transmission.

Plots of the interaction terms for each of the knowledge domains are shown in figure 3. Regarding MTCT, the wealth inequality*sex interaction plot shows that the odds of low knowledge of MTCT are higher in men at all levels of wealth inequality, and that among men these odds of low knowledge increase as wealth inequality increases. Among women, however, the odds of low knowledge increase as wealth inequality increases, until the highest wealth inequality category, at which the odds of low knowledge decrease. Furthermore, as absolute wealth rises, the odds of low knowledge of MTCT decrease among both men and women for each wealth quintile. Interestingly, however, men’s probability of low knowledge of MTCT does not decrease to the same level as that of women in the highest wealth quintile, which may be expected given that women may be more specifically targeted for MTCT interventions, for example as part of antenatal care.

The interaction plots for risk reduction show that women have a higher probability of low knowledge of risk reduction than men at any category of wealth inequality, and the likelihood of low knowledge rises for both sexes as wealth inequality rises. Similarly, concerning absolute wealth, women are more likely than men to have low knowledge of risk reduction in each wealth quintile, and this likelihood decreases as their wealth increases. Notably, the decrease in probability of low knowledge at each higher level of wealth is more pronounced in men than in women. Conversely, however, for knowledge of other modes of transmission, the probability of low knowledge decreases from the lowest to highest wealth inequality categories for both men and women, suggesting that high wealth inequality may impact access to some but not all domains of HIV-related knowledge.

Across all knowledge domains and in both men and women, the odds of low knowledge are decreased in the age group of 25–34 years old, with women having a higher probability of low knowledge in the general knowledge and risk reduction domains, but a lower probability of low knowledge of MTCT at each age group.

## Discussion

The study found higher odds of low HIV-related knowledge with decreasing wealth category, similar to a previous study indicating absolute poverty as a risk factor for HIV transmission.^8^ This finding suggests that a possible contributory factor giving rise to this relationship between poverty and HIV transmission, in particular in the Nigerian context, may be the comparatively low level of HIV-related knowledge among poorer strata of the population, leading to lower engagement in preventive behaviours and consequently a higher likelihood of HIV transmission in this group. The implications of this are that HIV-related knowledge may be a relevant factor influencing relationships between other sociodemographic risk factors and HIV transmission. As HIV-related knowledge is a targetable factor for strategic HIV prevention interventions, the low levels of HIV-related knowledge observed in the current study among the poor suggest that educational interventions to improve HIV-related knowledge should be preferentially targeted at marginalised population subgroups.

Although the authors hypothesised that wealth inequality may be a more significant predictor of HIV-related knowledge in Nigeria than absolute wealth, as studies in other Sub-Saharan African countries have indicated that high wealth inequality is associated with a higher risk of HIV transmission,^9–12^ the current study reports similar odds of low overall HIV-related knowledge across wealth inequality categories. However, importantly, when exploring the interaction effect of wealth inequality with sex, the finding that women have more than twice the odds of low overall HIV-related knowledge in comparison to men at all levels of wealth inequality suggests that women are more vulnerable to poor HIV-related knowledge, and by extension less able to advocate for preventive measures, under circumstances of wealth inequality. Moreover, the observation that the rise in the probability of low HK for women is more pronounced than that of men suggests that the effect of wealth inequality on access to HIV-related knowledge is influenced by gender. As suggested by our conceptual model, this indicates that the socioeconomic marginalisation experienced as a result of gender and wealth inequality represents a barrier to accessing HIV-related knowledge. As women may experience the combined effects of both gender-related and poverty-related marginalisation, they are less likely to have access to HIV-related information, and less likely to have the economic means and social empowerment to turn any acquired HIV-related knowledge into preventive health behaviours.

On the other hand, the fact that the probability of low HIV-related knowledge decreases at the highest wealth inequality category, becoming even lower for women than men in the same category, requires further exploration, particularly with regard to the prevailing HIV awareness and prevention programmes in high-wealth inequality states.

The fact that the decrease in probability of low HIV-related knowledge at each increasing national wealth quintile is more pronounced in men than in women (as was seen in the risk reduction knowledge domain and overall HIV-related knowledge interaction plots) suggests that women do not experience the protective effect of absolute wealth on HIV-related knowledge to the same extent as men, highlighting again that women face additional barriers to accessing HIV-related knowledge. These differences in results by sex indicate that apart from sex, the interaction effects of related covariates such as gender inequality or women’s empowerment on the relationship between HIV-related knowledge and wealth inequality should be explored, given that previous studies have highlighted women’s disempowerment,^31 32^ as well as the confluence of inequalities of gender and wealth,^20^ as significant social correlates of HIV infection in Nigeria.

Considering the observed low knowledge levels regarding the modes of MTCT of HIV in the overall sample, it is interesting to note that women displayed significantly lower odds of low HIV-related knowledge than men for the MTCT knowledge domain. This suggests that the observed low overall levels of MTCT knowledge may be attributable to men’s low knowledge. Considering that MTCT remains a significant source of new HIV cases in Nigeria, with an approximate 27.3% of pregnant HIV-positive women in Nigeria transmitting their infection to their child in 2014,^6^ the relatively high knowledge of MTCT among women suggests that although women are aware of the risks of perinatal HIV transmission, they continue to face barriers to adopting preventive measures. This may be due to being unable to advocate for preventive measures or acquire adequate prenatal care in the context of unequal gender dynamics with their male partner,^21^ or economic or geographical barriers to MTCT prophylaxis. Moreover, although women in this sample had higher knowledge of MTCT than men, women were significantly more likely than men to have poor knowledge of risk reduction measures, which indicates that MTCT educational interventions may have been successful at improving women’s knowledge in this area, but that the provision of specific educational programming for women regarding risk reduction should be increased, with an emphasis on female-driven preventive options (eg, pre-exposure prophylaxis).

Furthermore, given the significantly higher odds of low HIV-related knowledge among respondents with traditional religious beliefs, it is pertinent to consider HIV awareness programmes targeted at this group, and the appropriate adaptation of these programmes to traditional Nigerian religious and cultural values in order to improve programme acceptability.^33^ In addition, the drop in probability of low HIV-related knowledge in the age group of 25–34 years old compared with the high probability of low HIV-related knowledge in the group of 15–24 years old suggests the increased need for earlier HIV education among the younger population, particularly to ensure that HIV-related knowledge is high before sexual debut, rather than retrospectively. The analysis of the specific knowledge domains indicates that a particular focus on HIV risk reduction and prevention of MTCT programming is needed among this age group.

Lastly, those with low literacy levels being found almost twice as likely to have low HIV-related knowledge in comparison to literate respondents reiterates the need to target socioeconomically disadvantaged subgroups of the population in HIV-related educational programme, and strongly underlines the need to modify the medium of delivery of these interventions in order to ensure that they accommodate those with low literacy or the visually impaired (eg, through the use of verbal information dissemination rather than signage or written media).

Conclusively, as part of the new 2017–2021 National HIV and AIDS Strategic Framework, the Nigerian Agency for the National Control of AIDS has articulated its goal for 90% of vulnerable populations to adopt HIV risk reduction behaviours by 2021.^34^ In light of this, the identification of significant risk groups for low HIV-related knowledge in this study contributes to the evidence-informed targeting of interventions in order to meet this goal.

Significant limitations of this study, however, include first the comprehensiveness and predictiveness of the logistic regression model. As the model correctly classified only 68.1% of cases, care must be taken when interpreting ORs and subsequently drawing conclusions regarding risk groups for low HIV-related knowledge based on this model.

Moreover, although women’s empowerment has been identified as a relevant risk factor for HIV transmission in Nigeria, this could not be included as a potential predictor of HIV-related knowledge in the current model, as sufficient data on women’s empowerment indicators are not available in the male survey. The investigation of women’s empowerment as a predictor of HIV-related knowledge would however be relevant, particularly considering the fact that the interaction of wealth inequality and gender inequality has been shown to be a predictor of extramarital and transactional sex among women in Nigeria, thus predisposing them to a higher risk of HIV transmission.^20 21^ It would therefore be relevant to determine the role of HIV-related knowledge under these circumstances, and consequently its potential as a moderator of unsafe sexual behaviours in contexts of wealth and gender inequalities, as well as, ultimately, its value as a factor amenable to improvement for the reduction of HIV transmission in these contexts.

Moreover, in order to determine whether HIV-related knowledge is a significant predictor of actual risk of HIV infection, it would have been of interest to analyse individual HIV positivity in this sample as well; however, individual-level HIV testing data are not available in the 2013 NDHS. As relationships between health-related knowledge and subsequent health-related behaviours have been demonstrated,^35^ this study nonetheless provides a valuable evidence base for the targeting and adaptation of HIV-related educational interventions in Nigeria; however, the pertinence of future studies in the Nigerian context could be increased by an examination of the role of HIV-related knowledge as a predictor of actual HIV-related health behaviours and ultimately HIV infection.

Furthermore, the future investigation of wealth inequality as a direct predictor of actual HIV transmission (rather than HIV-related knowledge) in Nigeria is also relevant, given that subgroups of the poor who live in areas that are generally wealthy may be particularly likely to experience increased marginalisation, as such areas may be less likely to offer services or programmes that are targeted at, accessible to, or affordable for, its poorest residents. Therefore, poorer individuals living in areas of comparative wealth may, as a result of their social and economic exclusion, face significant barriers to accessing information, participating in preventive interventions or receiving treatment, and therefore ultimately be at higher risk for HIV transmission.

## Conclusion

Despite the limitations of this study in terms of the lack of individual-level HIV infection data, as well as the potential limitations of the accuracy of the model, the study fills a relevant knowledge gap regarding HIV-related knowledge in Nigeria, being the first study to examine in detail the sociodemographic determinants of HIV-related knowledge in the country, and the first to investigate wealth inequality as a significant predictor of HIV-related knowledge. The identification of risk groups for low HIV-related knowledge through this study will allow the more evidence-based design and targeting of HIV education and prevention programmes, and the underlining of wealth inequality as a barrier to accessing and acquiring HIV prevention information provides impetus for future studies in the Nigerian context to investigate the role of wealth inequality, rather than solely absolute poverty, as a predictor of actual HIV transmission risk. Ultimately, this will facilitate the improvement of our understanding of the marked heterogeneity in HIV prevalence seen across the country, and consequently the implementation of more effective preventive strategies among the most affected populations.
