# Supplementary material for: Wealth inequality as a predictor of HIV-related knowledge in Nigeria
Source: BMJ Glob Health. 2017 Dec 20;2(4):e000461. doi: 10.1136/bmjgh-2017-000461 (PMC5759704; doi:10.1136/bmjgh-2017-000461)
Supplement: Supplementary file 1 [file bmjgh-2017-000461supp001.pdf]

Appendix 1. Questions included in computation of HIV-related knowledge score

Question	Coding Yes	No
1. Has heard of AIDS	1	0
2. Knows a place to get HIV testing	1	0
3. Knows a source for condoms [a]	1	0
4. To reduce the risk of getting HIV: always use condoms during sex	1	0
5. To reduce the risk of getting HIV: have one sex partner only, who has no other partners	1	0
6. Can contract HIV from mosquito bite	0	1
7. Can contract HIV by sharing food with person who has AIDS	0	1
8. Can contract HIV by witchcraft or supernatural means	0	1
9. A healthy looking person can have HIV	1	0
10. HIV can be transmitted during pregnancy	1	0
11. HIV can be transmitted during delivery	1	0
12. HIV can be transmitted by breastfeeding	1	0

[a] Initially "does not know any source of condoms" in NDHS, re-defined as "knows a source of condoms" for ease of interpretation

NB: A further potentially relevant question, whether the respondent knows about the existence of "drugs to avoid HIV transmission to baby during pregnancy," was not included in the total score, due to the high number of missing cases for this question.
